# Supplementary material for: Birefringence-induced phase delay enables Brillouin mechanical imaging in turbid media
Source: Nat Commun. 2024 Jun 19;15:5202. doi: 10.1038/s41467-024-49419-2 (PMC11187154; doi:10.1038/s41467-024-49419-2)
Supplement: Supplementary file 1 — Supplementary Information [file 41467_2024_49419_MOESM1_ESM.pdf]

Supplementary Information and Figures

Birefringence-induced phase delay enables Brillouin mechanical imaging in turbid media

Giuseppe Antonacci[1*], Renzo Vanna[2], Marco Ventura[2], Maria Lucia Schiavone[3], Cristina Sobacchi[3,4], Morteza Behrouzitabar[2,5], Dario Polli[1,2,5], Cristian Manzoni[2**], Giulio Cerullo[2,5]

[1]Specto Photonics, Via Giulio e Corrado Venini 18, 20127 Milano, Italy
[2]CNR-Istituto di Fotonica e Nanotecnologie, CNR-IFN, Piazza Leonardo da Vinci 32, I-20133 Milano, Italy
[3]IRCCS Humanitas Research Hospital, via Manzoni 56, 20089 Rozzano (Milano)
[4]CNR-Istituto di Ricerca Genetica e Biomedica (CNR-IRGB), UOS di Milano, via Fantoli 16/15, 20138 Milano
[5]Dipartimento di Fisica, Politecnico di Milano, Piazza Leonardo da Vinci 32, I-20133 Milano, Italy
e-mail: *giuseppe@spectophotonics.com; **cristianangelo.manzoni@cnr.it

Supplementary Note 1 – Principles of the BIPD filter

A birefringent crystal is an anisotropic material characterized by a privileged direction named *optical axis*. In such material, the ordinary (o) polarization (i.e., normal to the optical axis) of light travels at speed c/n_o, while the extraordinary (e) polarization (i.e., parallel to the optical axis) travels at speed c/n_e, where n_o and n_e are the two refractive indexes and $\Delta n = |n_o - n_e| \neq 0$. At the output of the crystal, the light accumulates a phase retardation of

$$\varphi = 2\pi/\lambda \; L \; \Delta n \; ,$$

where λ is the central wavelength and L is the crystal thickness. When $\varphi = m\, 2\pi$ (with m an integer number), the *e* and *o* polarizations are in phase, so that the light has the same linear polarization as the input one. On the other hand, when $\varphi = (m+1/2)\, 2\pi$, the polarization of the output wave is rotated by 90°. Since the Rayleigh and the Brillouin signals have different wavelengths ($\Delta\lambda = | \lambda_0 - \lambda_B | \neq 0$), for a given crystal of birefringence Δn it is possible to design L so as to introduce a specific delay that results in a crossed polarization between the two fields at the output of the crystal. This condition is satisfied when the relative phase difference between the elastic (λ_0, Rayleigh) and inelastic (λ_B, Brillouin) signals is

$$\Delta\varphi = | \varphi_0 - \varphi_B | = \pi \; .$$

Assuming $\Delta n(\lambda_0) \sim \Delta n(\lambda_B)$, we have that the crystal length L is set by the following relationships as a function of wavelength

$$\mathrm{L(l)} = \frac{\mathrm{l}^2}{2\Delta \mathrm{l}} \frac{1}{\Delta n},$$

and frequency

$$\mathrm{L}(\nu) = \frac{\mathrm{c}}{2\nu_B} \frac{1}{\Delta n},$$

where ν_B is the frequency shift of the Brillouin scattered light. From the above equations we can obtain an expression for the filter Free Spectral Range

$$\mathrm{FSR} = 2\nu_B = \frac{\mathrm{c}}{L\Delta n}.$$

A linear polarizer (analyzer) with the transmission axis orthogonal with respect to the output linear polarization of the Rayleigh signal can therefore efficiently reject the elastic background light while fully transmitting the Brillouin signal.

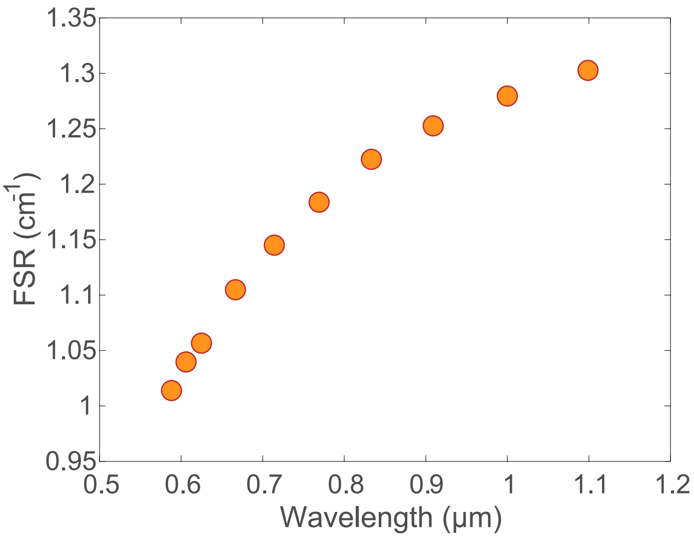

Supplementary Figure 1: **Measured FSR as a function of wavelength.** The BIPD filter was illuminated with broadband light from visible and NIR wavelengths and the transmitted signal was analysed by a FTIR spectrometer with 0.07 cm^{-1} spectral resolution. Changes in the measured FSRs across the spectrum are associated to variation in the YVO_4 birefringence at different wavelengths. Measurements demonstrate the broadband versatility of the filter that can be simultaneously used for a wide range of wavelengths.

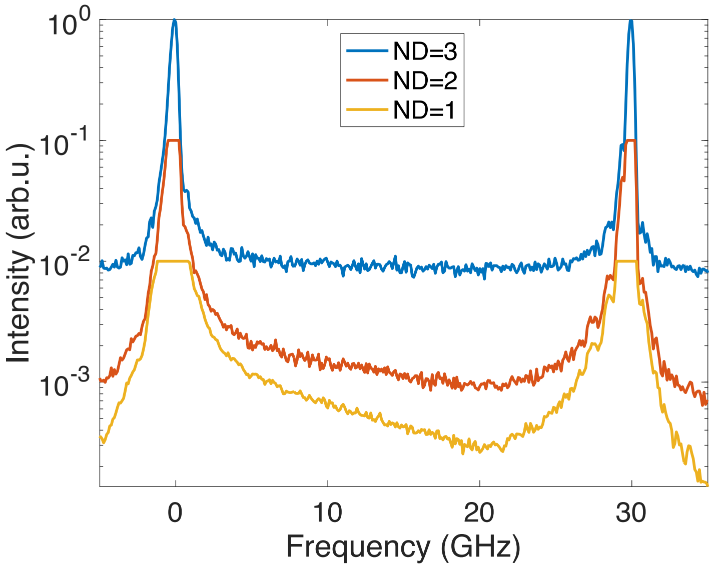

Supplementary Figure 2: **Spectral contrast of single-stage VIPA spectrometer.** The VIPA was illuminated with monochromatic light at different attenuation levels using calibrated neutral density (ND) filters to overcome the limited dynamic range of the CCD camera. The resulting transmitted profiles were measured at equal exposure time. Measured spectral contrast was ~30 dB, in agreement with the theoretical expectations. Fitting the non-saturated elastic peaks with a Lorentzian function, we measured a spectral resolution of FWHM = (515±20) MHz.

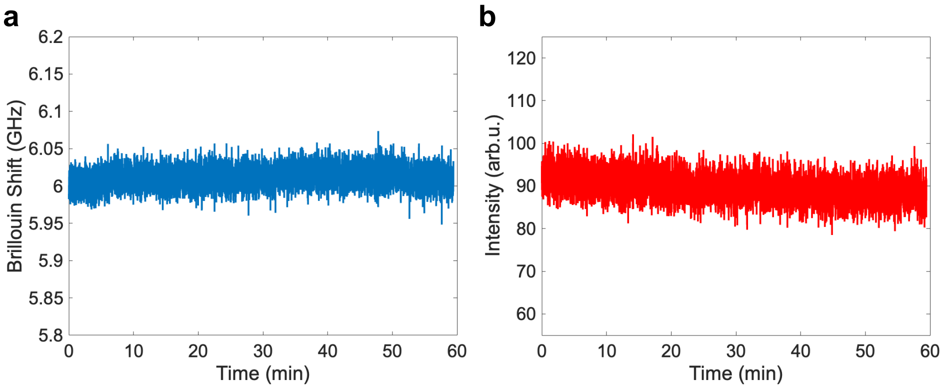

Supplementary Figure 3: **Sensitivity and SNR characterization. a,** Sensitivity of the single-stage VIPA spectrometer. The Brillouin spectrum of distilled water was acquired over 60 minutes with a pixel dwell time of 100 ms and fitted using a Lorentzian function. The instrumental sensitivity defined by the standard deviation of the measured frequency shifts is 14.5 MHz. **b,** Signal-to-noise ratio (SNR) characterization. A SNR=28.6±0.5 was obtained dividing the average Brillouin peak intensity by the standard deviation of the data points collected over the same time period.

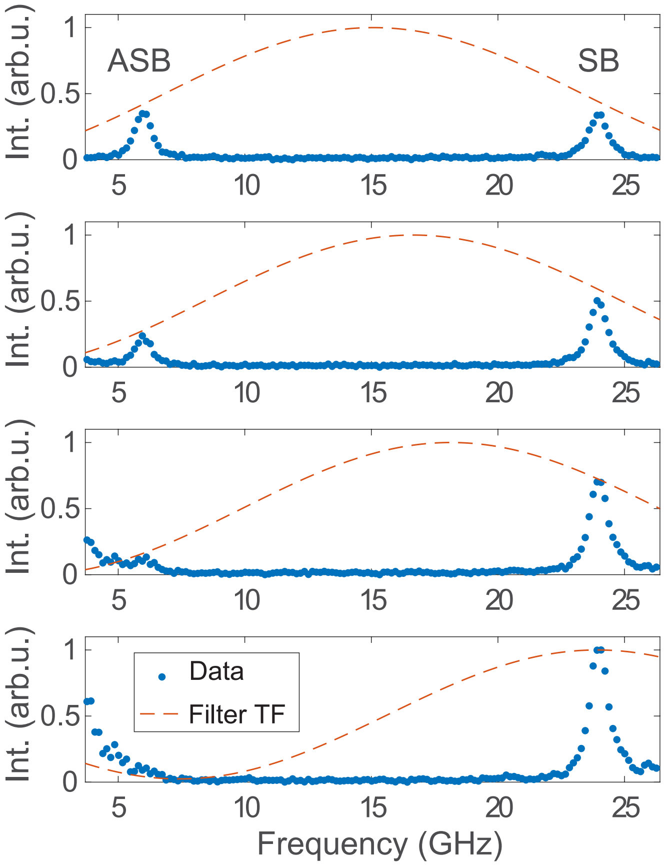

Supplementary Figure 4: **Spectral shift of the BIPD filter transmission function.** The spectrum of distilled water was acquired with the transmission maximum of the VIPA centred between two consecutive interference orders. The crystal was gradually tilted so as to induce a slight variation in the optical path, resulting in a shift of the filter transmission function (TF, red dashed line) along the FSR of the VIPA. As a consequence of the shift towards the higher VIPA interference order, the Anti-Stokes Brillouin peak (ASB) decreased in intensity till it was nulled by the filter. In parallel, the Stokes Brillouin peak (SB) gained ~4.5 dB in intensity when the transmission maximum was centred to the peak.

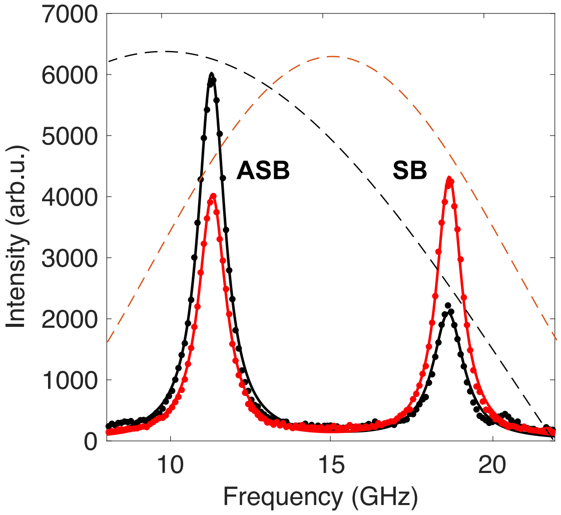

Supplementary Figure 5: **Characterization of peak changes in response to BIPD transmission.** Brillouin spectra of a polystyrene test sample acquired with the filter transmission function (dotted line) centred between the Stokes and anti-Stokes Brillouin peaks (red) and at the frequency of the anti-Stokes Brillouin peak (black). In the first case, the frequency shift and linewidth of the anti-Stokes Brillouin peak were measured by Lorentzian fitting to be $\nu_{ASB} = 11.33 \pm 0.05$ GHz and $\Delta\nu_{ASB} = 1.01 \pm 0.10$ GHz respectively, while the for the Stokes Brillouin peak we obtained $\nu_{SB} = -11.33 \pm 0.05$ GHz and $\Delta\nu_{SB} = 1.16 \pm 0.10$ GHz (fit error 1.4%, R>0.99). Similarly, the measured frequency shift and linewidth after translation of the filter transmission function were $\nu_{ASB} = 11.35 \pm 0.05$ GHz and $\Delta\nu_{ASB} = 1.02 \pm 0.10$ GHz for the anti-Stokes Brillouin peak, and $\nu_{SB} = -11.30 \pm 0.05$ GHz and $\Delta\nu_{SB} = 0.98 \pm 0.10$ GHz for the Stokes Brillouin peak (fit error 1.8%, R>0.99). Results demonstrate that the Brillouin spectrum is not affected by the transmission envelope of the BIPD filter.

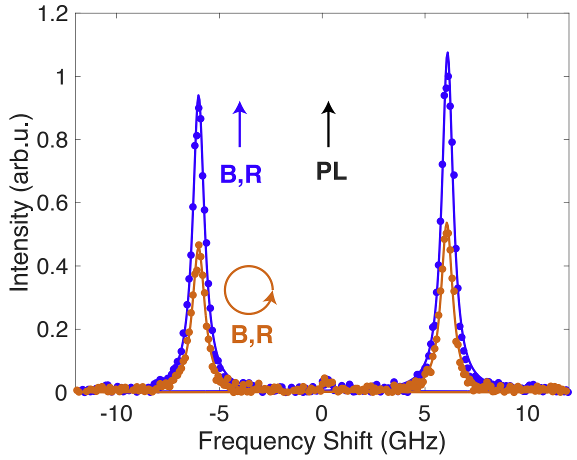

Supplementary Figure 6: **Response to sample birefringence.** While measuring the Brillouin spectra of water, a quarter wave plate was placed before the filter input polarizer to mimic the sample birefringence. With an appropriate choice of the wave plate optical axis, the polarization of the Brillouin (B) and Rayleigh (R) signals was turned to circular (red) from the original linear state (blue) parallel to the transmission axis (black) of the filter input polarizer. Apart from the expected extra insertion loss, a change in the input polarization did not affect the spectral shift of the Brillouin peaks ($\nu_B^{lin} = 6.04$ GHz; $\nu_B^{circ} = 6.06$ GHz) nor the ability of the filter to fully suppress the Rayleigh signal.

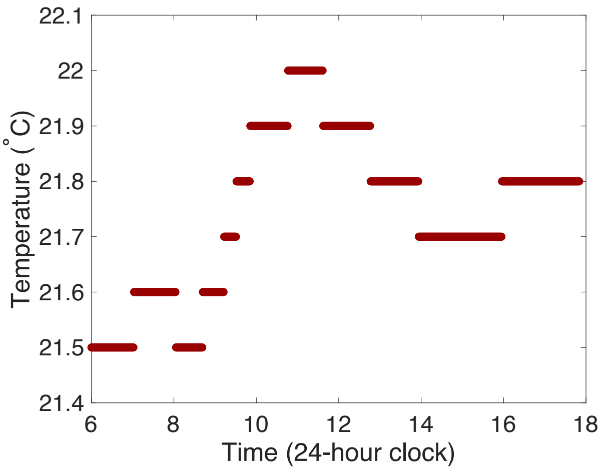

Supplementary Figure 7: **Environmental lab temperature variation.** Data has been recorded every second over a 12-hour interval using a digital temperature analyzer placed in proximity to the filter. Results show a maximum temperature variation of up to 0.5°C over the time of investigation.

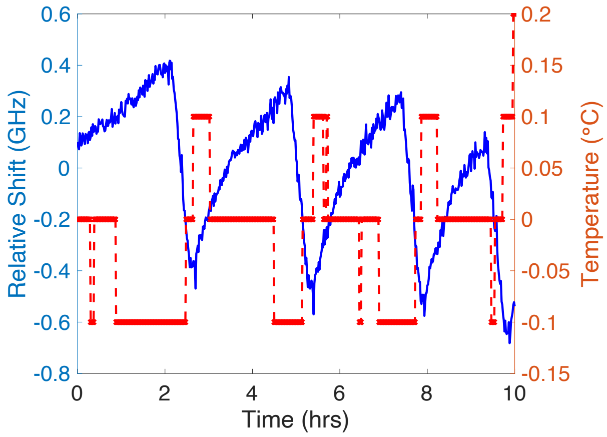

Supplementary Figure 8: **Laser frequency drift.** Monochromatic light from the laser was sent directly to the VIPA spectrometer and the resulting spectral profile was recorded for 10 hours. Values for the laser spectral drift (blue left y-axis) were obtained fitting the non-saturated Rayleigh peak for two consecutive interference orders of the VIPA. In parallel, the lab temperature was co-registered using a digital thermometer (red right y-axis). Results show a clear dependence of the laser stability on the ambient temperature, with an estimated laser drift of approximately 320±30 MHz for a temperature change of 0.1°C. In the time domain, we estimated a frequency drift of 4.8±0.5 MHz/min, reflecting our need to re-calibrate the BIPD filter with an active closed-loop control every 1-2 mins.

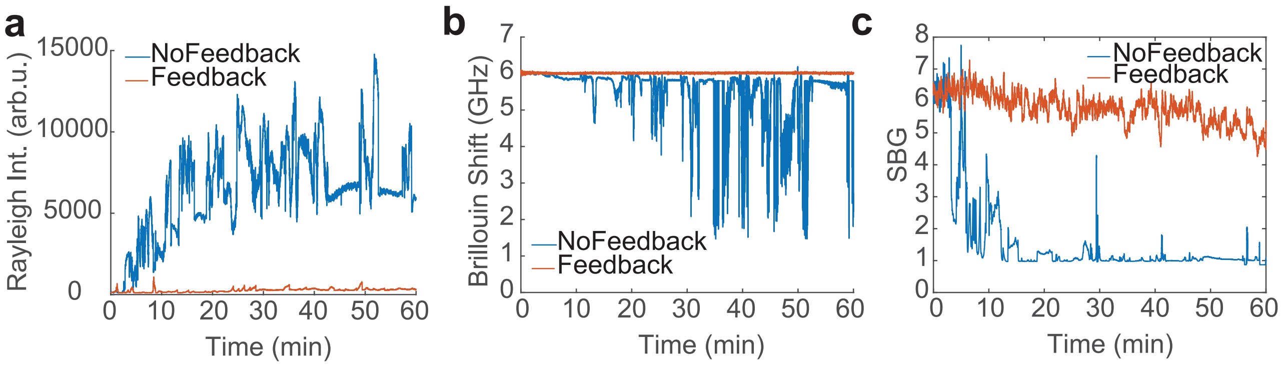

Supplementary Figure 9: **Closed-loop control for filter stabilization.** A direct comparison of the filter performance in terms of relative Rayleigh intensity (**a**), frequency shift (**b**) and signal-to-background (**c**) with and without feedback loop shows a significant enhancement of the filter stability over 60 mins. To align the filter at the laser wavelength, the liquid crystal was tuned by voltage sweep at 20 Hz with an incremental step of 1 mV, corresponding to a phase delay of ~0.5 mrad, and the transmitted Rayleigh intensity was measured sequentially using the CCD camera to determine the optimal phase retardance of the liquid crystal. This filter calibration process was iterated every 1 min resulting in almost negligible (~3%) extra contribution in the overall data acquisition time.

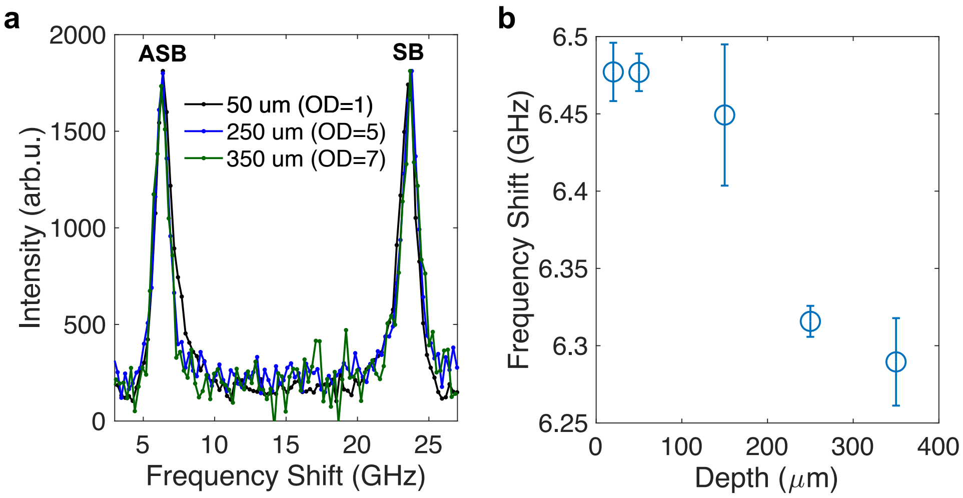

Supplementary Figure 10: **Brillouin spectra in the depth of pure milk. a.** Normalized Brillouin Stokes (BS) and anti-Stokes (ASB) peaks in the depth of 100% whole milk. Brillouin spectra were acquired with an integration time of 300 ms for z=50 μm, and 5 s for
z=250 μm and z=350 μm. **b**. Frequency shift measured by Lorentzian fitting as a function of depth. Values of the Optical Density (OD) were retrieved from the relationship $\mathrm{OD} = z \cdot \mu_e$, where z is the penetration depth and μ_e is the sum of the scattering and absorption coefficients. In the present study, we assumed $\mu_e = 20\ \mathrm{mm}^{-1}$ [1,2]. Although the Brillouin spectra do not exhibit noticeable shoulders, the frequency shift slightly decreased as a consequence of multiple scattering inside the turbid medium.

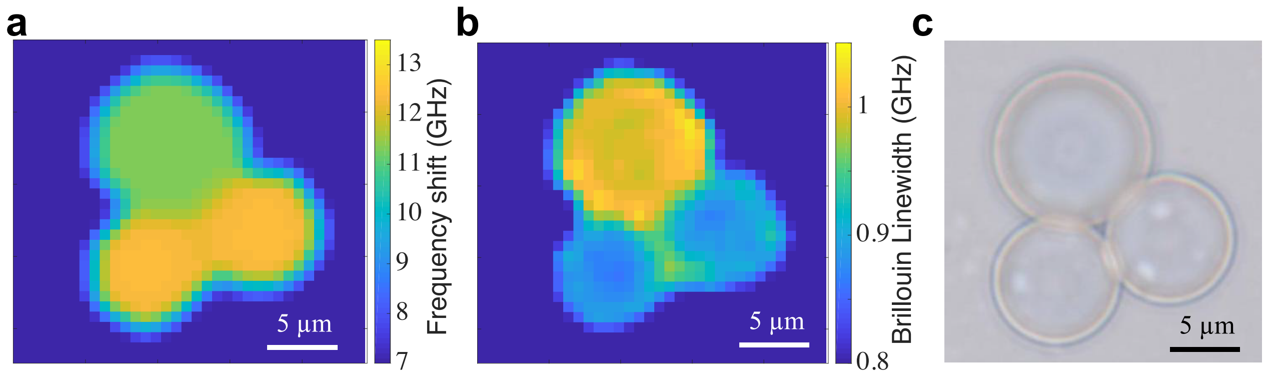

Supplementary Figure 11: **Brillouin maps of test beads.** Samples are a mixture of poly-methyl-methacrylate (PMMA) and polystyrene (PS) beads, respectively of 8 μm and 10 μm diameter, air-dried on a fused silica coverslip. Both frequency shift (**a**) and linewidth (**b**) show significant difference as a consequence of the different mechanical properties of the test beads which were spatially resolved by the imaging system. Associated bright-field image (**c**).

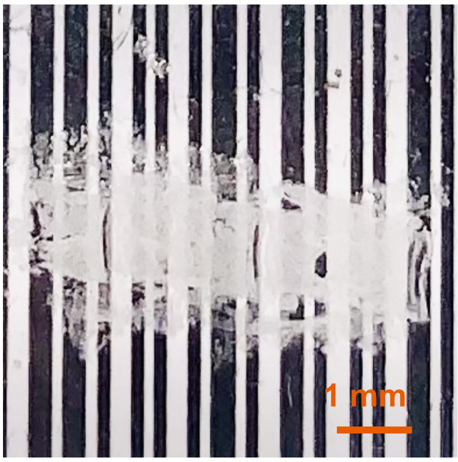

Supplementary Figure 12: **Unstained histological section of vertebra bone tissue.** The bone tissue was mounted on a microscope glass slice on top of a grid pattern. Connective bone tissues arguably represent the most challenging biological specimens to be measured in Brillouin microscopy as a consequence of the extreme turbidity as well as the high optical losses due to multiple scattering.

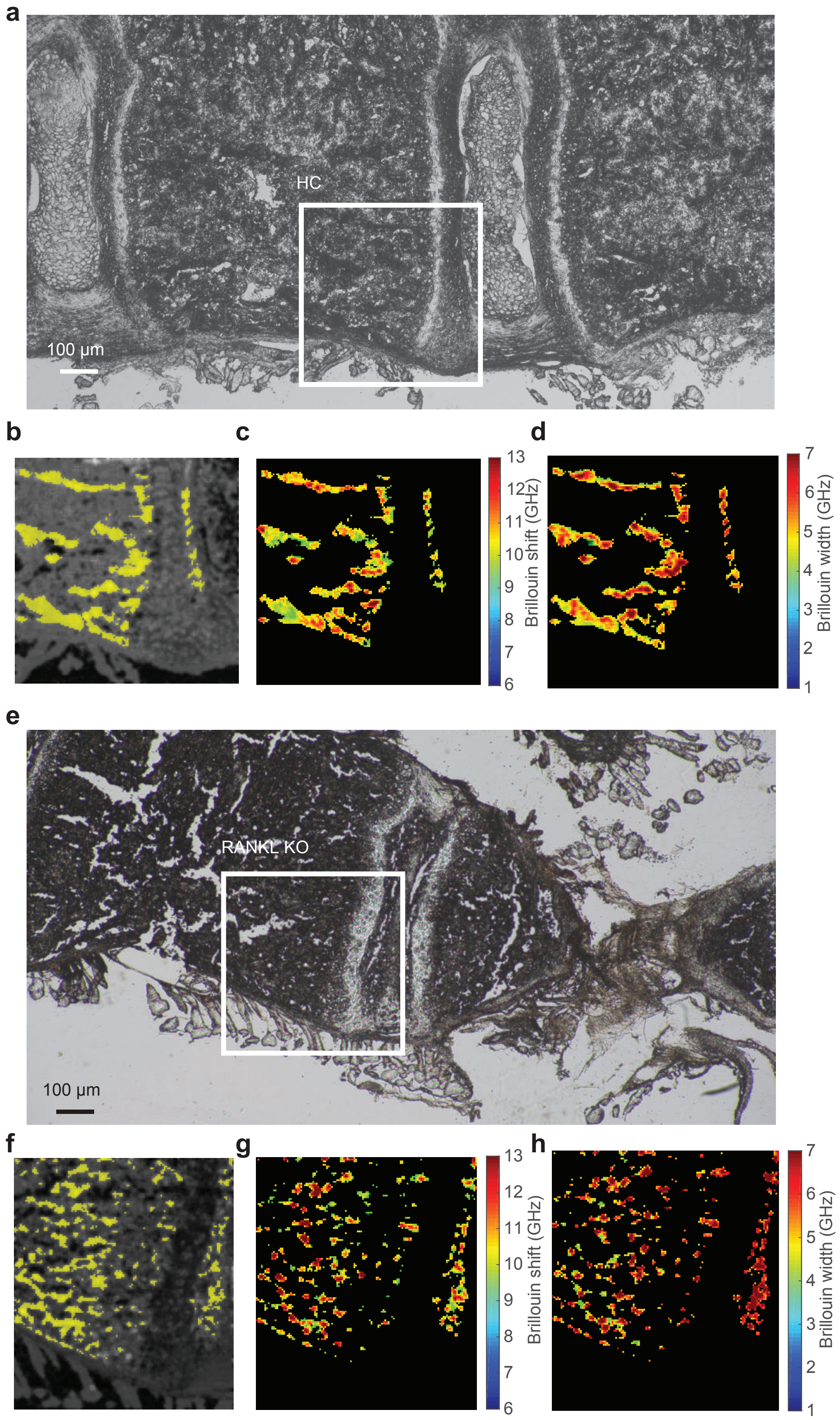

Supplementary Figure 13: **Image segmentation with Raman maps. a**, Bright-field image and selected region of interest (white box) of HC mice slice acquired with a 5x microscope objective. **b**, Superposition of normalized Brillouin map (grey) with mineralized apatite fraction (yellow) detected through spontaneous Raman imaging performed across the same region of interest and with equal scanning step size of 5 µm. **c**, Resulting Brillouin frequency shift and **d**, linewidth maps of bone content after segmentation with the Raman binary map. The same process was performed with the slice of the Rankl knockout mouse, as shown in the equivalent panels **e-f**.

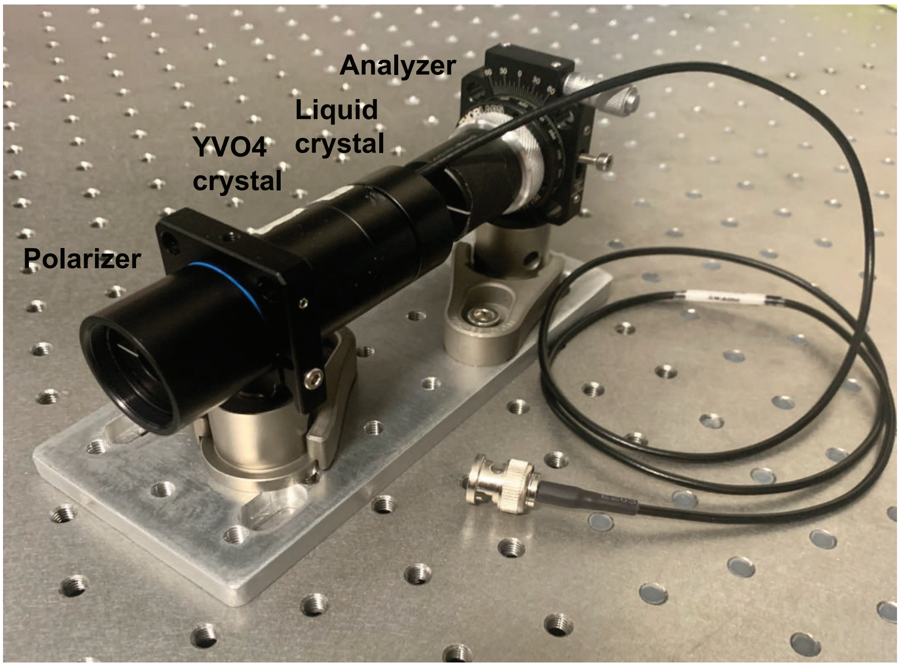

Supplementary Figure 14: **Image of the BIPD filter.** A full-wave liquid crystal retarder is used to tune the spectral transmission function of the filter. In the present configuration the filter has an overall dimension of 5x15 cm.

Supplementary References

[1] E. Berrocal, et al., Laser light scattering in turbid media Part I: Experimental and simulated results for the spatial intensity distribution, Opt. Express 15, 10649-10665 (2007)

[2] M.D. Waterworth, et al. Optical transmission properties of homogenised milk used as a phantom material in visible wavelength imaging, Australas Phys Eng Sci Med.;18(1), 39-44 (1995)
