# Supplementary material for: Birefringence-induced phase delay enables Brillouin mechanical imaging in turbid media
Source: Nat Commun. 2024 Jun 19;15:5202. doi: 10.1038/s41467-024-49419-2 (PMC11187154; doi:10.1038/s41467-024-49419-2)
Supplement: Supplementary file 3 — Description of Additional Supplementary Files [file 41467_2024_49419_MOESM3_ESM.pdf]

Description of Additional Supplementary Files

File Name: Supplementary Movie 1

Description: Elastic background suppression by BIPD filtering.
